# Mblk-1 Transcription Factor Family: Its Roles in Various Animals and Regulation by NOL4 Splice Variants in Mammals

**DOI:** 10.3390/ijms18020246

**Published:** 2017-01-25

**Authors:** Seika Takayanagi-Kiya, Taketoshi Kiya, Takekazu Kunieda, Takeo Kubo

**Affiliations:** 1 Division of Life Sciences, Graduate School of Natural Science and Technology, Kanazawa University, Kanazawa, Ishikawa 920 1192, Japan; kiya@staff.kanazawa-u.ac.jp; 2 Department of Biological Sciences, Graduate School of Science, The University of Tokyo, Bunkyo-ku, Tokyo 113 0033, Japan; kunieda@bs.s.u-tokyo.ac.jp (T.K.); stkubo@bs.s.u-tokyo.ac.jp (T.K.)

**Keywords:** honeybee, transcription factor, Mblk-1, E93, MBR-1, Mlr1, LCoR, NOL4, transcription cofactor, splice variants

## Abstract

Transcription factors play critical roles in regulation of neural development and functions. A transcription factor Mblk-1 was previously reported from a screen for factors possibly important for the higher brain functions of the honeybee. This review first summarizes how Mblk-1 was identified, and then provides an overview of the studies of Mblk-1 and their homologs. Mblk-1 family proteins are found broadly in animals and are shown to affect transcription activities. Studies have revealed that the mammalian homologs can interact with several cofactors and together regulate transcription. Interestingly, a recent study using the mouse homologs, Mlr1 and Mlr2, showed that one of their cofactor proteins, NOL4, have several splice variants with different effects on the transactivation activities of Mlr proteins. These findings suggest that there is an additional layer of the regulation of Mblk-1 family proteins by cofactor splice variants and provide novel insights into our current understanding of the roles of the conserved transcription factor family.

## 1. Introduction

Despite the wide variety of behaviors exhibited by various animal species, some of the genetic pathways used to achieve brain functions are highly conserved across the animal kingdom [1,2,3]. For example, molecular pathways involving the transcription factor cre-binding protein (CREB) are highly conserved through evolution [1]. Additionally, studies have shown that transcriptional regulation of gene expression is vital for various neuronal functions. As for CREB, it plays critical roles in learning and memory from nematodes to mammals [1,2].

A novel transcription factor Mblk-1 was identified from a transcriptomic non-hypothesis-driven approach focusing on the honeybee higher brain centers as a candidate that can be potentially important for the highly social behaviors of the honeybee [4]. Mblk-1 was found to be conserved widely in the animal kingdom, and studies on Mblk-1 family proteins described important roles of the protein in both invertebrates and vertebrates. Here we review the process of identification of Mblk-1 and then the functions of Mblk-1 family transcription factors by covering studies from *C. elegans* to mammals. We then describe recent studies where specific cofactors and their splice variants were shown to play important roles in regulation of the functions of the Mblk-1 family transcription factors. Finally, we discuss future perspectives including thoughts of how splice variants can contribute to the regulation of Mblk-1 proteins.

## 2. Honeybees Exhibit Highly Organized Social Behavior and Have Enlarged Mushroom Bodies (MBs)

European honeybee, *Apis mellifera* L., is an insect that organizes a highly ordered society. Their colony consists of a single queen, 10,000–40,000 workers that are non-reproductive females, and 300–1500 males called drones [5,6]. Workers perform various tasks to maintain the colony depending on their ages and colony demands. The most intriguing feature is a well-known “dance-language”, with which the foraging workers are able to transmit the location of food sources to nestmates through symbolic waggle dances, making the honeybee a fascinating animal model to analyze symbolic language and sociality [7].

What molecular and neural mechanisms enable honeybees to organize highly ordered society, conduct a variety of tasks throughout their life, and communicate using the symbolic dance language? As an approach to answer this question, we focused on the mushroom bodies (MBs) of the honeybee brain. The MBs are famous as important brain regions of olfactory learning in the vinegar fly, *Drosophila melanogaster* [8]. In *Drosophila melanogaster*, olfactory information and valence signal converges in the MBs, where association between odor and value is established. In honeybees, the MBs receive multimodal information and play important roles in higher-order learning [9,10]. The MB neurons also exhibit structural plasticity, where neurite develops in foragers by the sensory experience derived from foraging [11,12]. In addition, activity mapping by immediate early genes, neural activity markers, revealed that a subtype of MB neurons is active in dancers [13], and this neural activity is related to visual inputs and foraging experience [14]. Furthermore, the MBs are highly developed in honeybees compared to other insects [15]. The honeybee brain consists of 1,000,000 neurons and the MBs have 300,000 neurons [16], whereas the brain of *Drosophila melanogaster* consists of 100,000 neurons and the MBs have 2000 neurons [17]. Additionally, the MBs are composed of two calyces per hemisphere in honeybees, but flies have a single calyx in each hemisphere. This structural and functional expansion of MBs in the honeybee brain could underlie why honeybees can perform diverse repertories of behavior and have highly sophisticated abilities.

## 3. Mblk-1 Is Preferentially Expressed in Honeybee MBs

**Figure 1:**
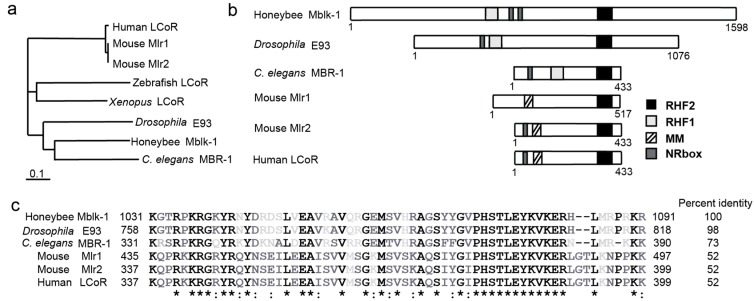
Mblk-1 family proteins are conserved among animals. (**a**) A phylogenetic tree of Mblk-1 family proteins. Generated by ClustalX2 using the neighbor-joining method; (**b**) Structures of Mblk-1 family proteins. RHF1, RHF2: helix-turn-helix DNA binding motifs. MM: mammalian Mlr motif; NR box: nuclear receptor binding motif. Adapted with permission from [23]; (**c**) Amino acid sequence alignment and the percent identities of the conserved RHF2 motifs from Mblk-1 family proteins. * Indicates a fully conserved residue; : indicates conservation between amino acids with strongly similar properties. Generated using Clustal Omega [24].

Based on the hypothesis that elaborated MBs are related to the social behaviors and/or highly advanced brain functions of honeybees, we conducted a transcriptomic screen to identify genes preferentially expressed in the MBs [4,18,19,20]. From this screen, we found that genes related to the calcium-signaling pathway, such as CaMKII, IP_3_ receptor, and ryanodine receptor, are preferentially expressed in the MBs. Since these genes are important for higher brain functions [1,21,22], the results suggested that the MBs of honeybees highly express genes related to those brain functions. Besides these well-known genes, we identified a gene expressed specifically in a subtype of MB neurons called large-type Kenyon cells, and named it Mblk-1 (mushroom body large-type Kneyon cell-specific protein-1) [4]. The protein is conserved widely among animals (Figure 1a–c). Two well conserved motifs, RHF1 and RHF2, exhibit certain similarities to other DNA-binding proteins, suggesting transcriptional regulatory activity of Mblk-1. Thus, Mblk-1 was identified as a novel transcription factor that may play important roles in higher brain functions.

The DNA sequence to which Mblk-1 preferentially binds was identified from a binding site selection assay, where random short double-stranded DNA were screened for its affinity to Mblk-1 in vitro [25]. Mobility shift assay also revealed the selective binding of Mblk-1 to the identified 22 base-pair sequence, termed MBE (Mblk-1 binding element). Further, Mblk-1 upregulates transcription of reporter genes in an MBE-dependent manner when expressed in cultured cells. Additionally, its transactivation activity is modulated by the Ras/MAPK pathway [26]. Interestingly, expression of MAPK is upregulated in the brain of foragers than in that of nurse bees [27]. These results imply the possibility that foraging experience induces MAPK expression, leading to modulation of Mblk-1 transcription activity and plastic changes in MB neural function. Specific functions of Mblk-1 in the honeybee brain, such as the downstream targets and the exact timing of expression, are yet to be characterized.

## 4. *Drosophila* Homolog E93 Is Involved in Regulation of Development and Morphogenesis

Proteins with high homology in the DNA binding motifs of Mblk-1 are found widely in the animal kingdom (Figure 1b). One of the homologs in insects is a *Drosophila* protein E93, functions of which have mostly been studied from the view of its roles in the regulation of development and metamorphosis [28]. E93 was initially reported as a gene actively transcribed during the prepupal stage in *Drosophila*. Its expression was found in several tissues including the salivary gland and the central nervous system, and was induced in response to 20-hydroxy-ecdysone (20E), the insect steroid hormone that regulates molting and metamorphosis. Another study revealed that its expression is upregulated in cells that undergo apoptosis, and is required to promote proper programmed cell death during metamorphosis [29]. Additionally, it was shown that E93 mutants have defects in expressing 20E-induced as well as apoptosis-related genes, consistent with the idea that E93 regulates transcription of those genes.

A more recent report showed that functions of E93 are also required for adult morphogenesis during the pupal stage [30], suggesting that it has roles in development independent of the apoptosis pathways. Interestingly, the function of E93 to promote adult morphogenesis is conserved between holometabolous (*Drosophila melanogaster* and *Tribolium castaneum*) and hemimetabolous (*Blattella germanica*) insects, suggesting that it may function as a universal regulator of adult body patterning in insects [31]. Additionally, E93 has been reported to affect gene expression in neurons. RNAi knockdown of E93 in olfactory sensory neurons caused a lack of expression of Or47b [32], an odorant receptor involved in pheromone perception and proper courtship behavior of males [33,34]. The nematode *Caenorhabditis elegans* homolog MBR-1 was also shown to have neuronal functions, where it is required for the pruning of specific neurites that occur during larval development [35,36]. Importantly, it was also suggested that MBR-1 is required for olfactory plasticity in adult animals [36], providing support for the possible involvement of Mblk-1 family proteins in behavioral plasticity.

**Figure 2:**
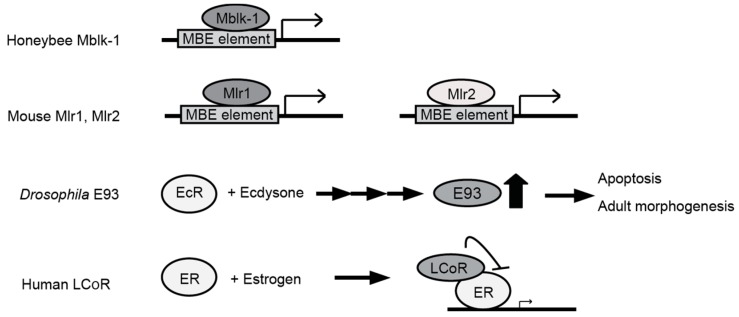
Reported functions and regulations of Mbk-1 family proteins. Honeybee Mblk-1 binds to MBE sequence in vitro, and activates transcription of the downstream gene. Mouse Mlr1 and Mlr2 also show MBE-dependent transactivation activity. Expression of *Drosophila* E93 is upregulated by ecdysteroid in pupal stages, likely under the control of ecdysone receptor (EcR) complex. E93 is required for expression of genes related to apoptosis and adult body patterning. Human LCoR interacts with estrogen receptor (ER) and suppresses transcription of ER target genes, but recent studies suggest that it may also act to enhance transcription of several target genes.

An important aspect pointed out from the studies in *Drosophila* is the effect of steroid hormone 20E on the expression and transcription activities of E93 [29,30]. A current model predicts that transcription of Mblk-1 family proteins is upregulated by the ecdysone receptor directly or indirectly, and they interact with other co-factors to regulate the downstream gene expression [29] (Figure 2). The actual mode of interaction of the proteins and possible co-factors interacting with Mblk-1 proteins in insects remain to be examined.

The remarkable conservation of the nuclear recognition motif NR box (a motif involved in interaction with nuclear receptors [37]) in Mblk-1 family proteins among animals raises a possibility that the roles of Mblk-1 proteins are conserved from invertebrates to vertebrates to a certain extent, where they regulate steroid-induced genes by interacting with other nuclear receptors involved in the molecular pathways. Indeed, mammalian homologs of Mblk-1 have been shown to be involved in the steroid hormone-related pathways, as described below.

## 5. LCoR, the Human Homolog of Mblk-1, Interacts with Multiple Cofactors and Modulates Transcription Activities

Mammalian homologs of Mblk-1 have one helix-loop-helix domain with high homology to RHF2 (Figure 1b,c). The human homolog is named ligand-dependent corepressor (LCoR), which has the conserved DNA-binding motif, the NR box, and a nuclear localization signal, and was first identified as an interactor of estrogen receptor α (ERα) from a yeast two-hybrid screen [38]. ERα is a well-known nuclear receptor that acts as a transcriptional regulator upon binding of female steroid hormone estrogen [39]. LCoR was shown to be expressed wildly in fetal and adult tissues of humans, including the cerebellum and corpus callosum in the brain [38]. In cell cultures, LCoR showed binding to ERα in the NR box—and in an estradiol-dependent manner. Furthermore, expression of LCoR suppressed transactivation activity of ERα, suggesting its roles in hormone-dependent regulation of transcription as a co-repressor.

LCoR was also shown to directly interact with androgen receptor (AR), a nuclear hormone receptor activated by the male steroid hormone [40]. Similar to its effect on ERα, LCoR suppresses the transactivation activity of AR. However, unlike ERα, the NR box of LCoR was dispensable for this effect, suggesting that the interaction of LCoR with ERα and AR occur in different manners. More recently, LCoR was shown to interact with several other proteins. It interacts with another transcription factor Krüppel-like Factor 6 (KLF6), and regulates expression of some of its target genes including cyclin-dependent kinase inhibitor CDKN1A [41]. Additionally, histone deacetylase 3 (HDAC3) and HDAC6 interacts with LCoR, implying that LCoR may regulate transcription together with these proteins [38,42]. LCoR also interacts with another corepressor KRAB-associated protein-1 (KAP-1) to form a complex with a transcription factor ZBRK1, and suppress expression of genes including fibroblast growth factor 2 (FGF2) [43]. FGF2 expression is known to be suppressed in breast cancer cells [44], and the suppression by LCoR and its interacting factors promotes the survival of both malignant and non-malignant breast epithelial cells [41]. These findings highlighted the possible involvement of LCoR and its interacting factors in the suppression and development of cancer.

Though studies of LCoR have mainly reported its function as a transcriptional co-repressor, it can function to promote expression at least for some genes [42,45]. LCoR ablation in cultured cells by siRNA expression caused increased expression of some ERα-target genes such as IGFBP1, but it also caused decrease (e.g., CYP26B1) or had no effect (e.g., pS2) on several target genes [43]. These observations suggest that the role of LCoR on transcription activity is different from gene to gene.

Taken together, the studies suggest important roles of LCoR in regulating hormone-induced pathways and the development of cancer. From its expression from fetal stages to the adulthood, LCoR likely functions throughout the human development. On the other hand, its roles in the brain still remain elusive with little functional studies.

## 6. The Mouse Homologs Mlr1 and Mlr2 Have Transactivation Activities and Show Different Expression Patterns

A series of studies on the mouse homologs of Mblk-1 and their interacting factors also suggest that they can have different effects on transcription activity depending on the context. The two mouse homologs, termed Mlr1 (Mblk-1 related protein-1) and Mlr2, encode proteins with the conserved helix-loop-helix DNA binding domain RHF2, and Mlr2 has 98% amino acid sequence homology to the human homolog LCoR [46]. Mlr1 has the NR-box, whereas Mlr2 lacks the motif, which suggests that the two proteins may have different interacting partners. The two genes have different expression patterns, where Mlr1 is expressed strongly in the testis and more weakly in the kidney, liver, and heart, whereas Mlr2 is expressed in tissues including kidney, liver, and heart, but not testis. Expression in the brain was low for both genes, though it does not exclude the possibility that they are expressed in a spatially and/or temporally controlled manner. Examination of transcription activities by luciferase assay revealed that Mlr1 and Mlr2 both recognize MBE element, the 22-bp element that Mblk-1 binds to, and enhance transcription, similar to what was observed for Mblk-1. These results suggest that the function of Mblk-1 family proteins, especially of the RHF2 motif, can be conserved from insects to mammals.

## 7. Mlr1, Mlr2 Interact with Multiple Cofactors Including a Conserved Nucleolar Protein NOL4

Functions of transcription factors are often modified by their interacting partners [47,48], and studies of LCoR showed that it interacts with multiple proteins as described above. However, little is known about the roles of Mblk-1 family proteins in the nervous system. In search for interacting factors of Mlr1 and Mlr2 in the mouse brain, we performed a yeast-two-hybrid screen and identified possible interacting proteins [23]. Nucleolar protein 4 (NOL4) and protein C20orf112 homolog, which consists of a protein family with similar primary structures, showed strong interaction with Mlr1. Additionally, Myosin-5B, CtBP1, CtBP2, and slc15a2 were identified as minor interactors. For Mlr2, CtBP1 was the main interactor, and NOL4, mastrin, and SNX16 were minor interactors. CtBP proteins are known as transcriptional corepressors [49], and human CtBP1 and CtBP2 were previously reported to interact with LCoR, the human homolog of Mblk-1, and affect transcriptional activities [38].

**Figure 3:**
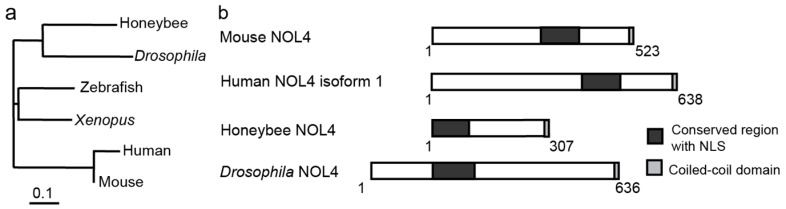
A conserved protein NOL4 has multiple splicing variants that differentially regulate the transcription activities of Mlr1 and Mlr2. (**a**) A phylogenetic tree of NOL4 family proteins. Generated by ClustalX2 using the neighbor-joining method; (**b**) Structures of NOL4 family proteins. Conserved regions with NLS and the coiled-coil domain are designated. Adapted with permission from [23].

Homologs of NOL4 are found widely among animals (Figure 3a,b). In humans, NOL4 is expressed specifically in the fetal and adult brain and testis, and locates to the nucleoli in cultured cells [50], but their molecular functions remained uncharacterized. Zebrafish has two homologs of NOL4, called Znol4la and Znol4b, which has high amino acid sequence homology to the human NOL4 [51]. Zebrafish embryo expresses Znol4b strongly in the central nervous system including the spinal cord and hindbrain, suggesting that it may have neuronal functions.

## 8. Splicing Variants of NOL4 Differentially Affect the Transactivation Activities of Mlr1 and Mlr2

**Figure 4:**
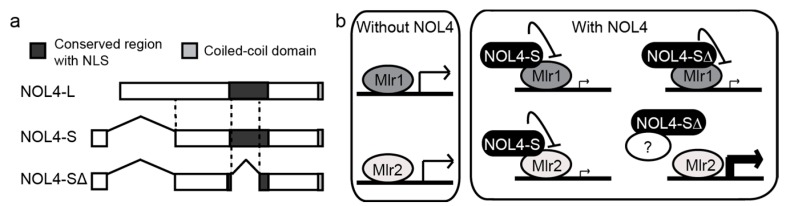
Mouse NOL4 splicing variants have different functions. (**a**) Schematics of the splicing variants of mouse NOL4. N-terminal region in NOL4-L is spliced out in NOL4-S and NOL4-SΔ. Major part of the conserved region containing NLS is spliced out in NOL4-SΔ. Adapted with permission from [23]; (**b**) A model describing how Mlr1 and NOL4 regulate transcription. Left panel: Without NOL4, Mlr1, and Mlr2 show transactivation activities. Right panel: When NOL4 variants are present, NOL4-S directly binds to Mlr1 and Mlr2, and inhibits the transactivation activity. NOL4-SΔ does not directly bind to Mlr2, but it may bind to another cofactor (represented by “?” in the figure), possibly CtBP proteins which suppress the activity of Mlr2, and thereby indirectly increase the transcription activity.

We characterized that murine NOL4 has three splicing variants with different structures (Figure 4a), termed NOL4-L, NOL4-S, and NOL4-SΔ [23]. NOL4-L and NOL4-S have a conserved nuclear localization sequence (NLS), whereas NOL4-SΔ does not. All of the variants have a conserved coiled-coil domain at the C-terminus. Interestingly, the variants have differential binding properties to Mlr1 and Mlr2, where NOL4-S showed strong binding to both Mlr1 and Mlr2 by GST pull-down assay but NOL4-SΔ showed binding only to Mlr1. Moreover, NOL4-S and NOL4-SΔ have different effects on the transactivation activity of Mlr1 and Mlr2. Specifically, NOL4-S suppressed the transactivation activity of both Mlr1 and Mlr2, whereas NOL4-SΔ only suppressed Mlr1 and increased the level of transcription when expressed with Mlr2. Suppression of the transactivation activity of Mlr1 may be caused by conformational changes of the protein by direct binding of NOL4-S (Figure 4b). Since no direct binding was observed between Mlr2 and NOL4-SΔ, the increase in transcription may be explained by an indirect mechanism. For example, NOL4-SΔ may bind to CtBP1, which interacts with Mlr2, and sequester CtBP1 from the transcription site, which can result in suppressing the inhibition of transcription by CtBP1.

## 9. Future Perspectives

Much knowledge on the Mblk-1 family proteins has accumulated since their initial identification, but many questions remain unanswered. For example, the degree of conservation of the protein functions among animals remains unclear. Honeybee Mblk-1 as well as Mouse Mlr1 and Mlr2 act as transcription factors with transactivation activities, but can *Drosophila* E93 and human LCoR do the same? LCoR was found to regulate steroid-hormone-induced gene expression, but does it also interact with the MBE sequence when affecting transcription? Where on the genome do Mblk-1 proteins bind to in vivo in different tissues of animals? Answering these questions will further deepen our understanding of how the transcription regulatory mechanisms have evolved.

Neuronal functions of Mblk-1 also remain elusive. Up to this point, studies of Mblk-1 family proteins focused mostly on developmental functions of the proteins. However, our initial study of Mlbk-1 detected strong expression of Mblk-1 in adult MBs in the honeybee brain [4], implying that it can have functions in the mature nervous system. Additionally, the *Drosophila* homolog E93 was reported to be required for expression of at least one olfactory receptor in the adult [32] and proper courtship behavior. In addition, mammalian homologs are found to be expressed in the adult brain. An intriguing hypothesis is that the transcription factors switch their roles after development and are involved in higher brain functions such as learning, memory, and/or social behaviors in adults. Increasing evidence suggests that Mblk-1 family proteins can contribute to the regulation of transcription in a context-dependent manner in various animals, depending on developmental stages and different interacting cofactors. In the future, tissue- and developmental stage-specific modification of gene expression will be informative to reveal the neuronal function of Mblk-1 family proteins in adults. The conservation of Mblk-1 family proteins and their cofactors raises a possibility that there is a transcriptional mechanism widely used by animals to control neural development and function by recruiting a common set of proteins.

Another interesting question is whether the differential regulation of Mblk-1 family proteins by NOL4 splicing variants is conserved from invertebrates to mammals. Other studies have also reported differential regulation of transcription factors by cofactor splice variants. For example, amplified in breast cancer 1 (AIB1), a human nuclear receptor coactivator, was shown to have two splice variants with different subcellular localizations and different levels of effectiveness in the activation of estrogen-dependent gene transcription [52]. Additionally, three isoforms were identified for Steroid Receptor Coactivator-1 (SRC-1), which regulates gene expression in response to thyroid hormone in humans, and were shown to have different transactivation efficiencies [53]. Whether these splice variants are conserved across species remains to be investigated. Since NOL4 is conserved among animals, it is possible that there are evolutionarily conserved mechanisms involving the transcription cofactors. Examining the precise expression patterns of cofactors and their splicing variants will further elucidate where and when the specific transcriptional regulations take place in animals. The transactivation activity by Mblk-1 family proteins can be under tight controls of the spatially and temporally controlled expression of cofactors and their specific splicing events.
